# Biomimetic Hydrogel Strategies for Cancer Therapy

**DOI:** 10.3390/gels10070437

**Published:** 2024-06-30

**Authors:** Awatef M. Alshehri, Otto C. Wilson

**Affiliations:** 1 Department of Biomedical Engineering, The Catholic University of America, Washington, DC 20064, USA; 2 Department of Nanomedicine, King Abdullah International Medical Research Center (KAIMRC), King Saud bin Abdelaziz University for Health Sciences (KSAU-HS), Ministry of National Guard-Health Affairs (MNGHA), Riyadh 11426, Saudi Arabia; alshehriaw@kaimrc.edu.sa

**Keywords:** biomimetic, cancer metastases, hydrogel, cancer therapy

## Abstract

Recent developments in biomimetic hydrogel research have expanded the scope of biomedical technologies that can be used to model, diagnose, and treat a wide range of medical conditions. Cancer presents one of the most intractable challenges in this arena due to the surreptitious mechanisms that it employs to evade detection and treatment. In order to address these challenges, biomimetic design principles can be adapted to beat cancer at its own game. Biomimetic design strategies are inspired by natural biological systems and offer promising opportunities for developing life-changing methods to model, detect, diagnose, treat, and cure various types of static and metastatic cancers. In particular, focusing on the cellular and subcellular phenomena that serve as fundamental drivers for the peculiar behavioral traits of cancer can provide rich insights into eradicating cancer in all of its manifestations. This review highlights promising developments in biomimetic nanocomposite hydrogels that contribute to cancer therapies via enhanced drug delivery strategies and modeling cancer mechanobiology phenomena in relation to metastasis and synergistic sensing systems. Creative efforts to amplify biomimetic design research to advance the development of more effective cancer therapies will be discussed in alignment with international collaborative goals to cure cancer.

## 1. Introduction

Cancer is a complex disease characterized by the uncontrolled growth and proliferation of cells [1]. Cancer cells have the ability to evade normal regulatory mechanisms, resulting in the formation of self-propagating tumors with the potential to metastasize and spread to other parts of the body [2]. Cancer cell biology covers a wide range of phenomena including genetic mutations, tumor heterogeneity, cancer stem cells, mechanobiology effects, and metabolic adaptations [1,2]. Scientists aim to uncover the complexities of cancer at the cellular and sub-cellular levels to create specifically tailored treatments that can successfully block tumor progression and improve patient outcomes.

One of the remarkable features of cancer involves its ability to adapt to different environments and develop resistance to treatments [3]. There are numerous challenges that impact the effectiveness of cancer therapy and patient outcomes such as the rising cost of chemotherapy, limited accessibility to treatment options, and the resistance of cancer cells to drugs [4]. The insufficient delivery of both conventional and nanoparticle-based therapies can lead to problems such as rapid drug clearance and drug resistance [5]. The mononuclear phagocytic system (MPS) targets nanoparticles and poses obstacles to the use of nanomaterials that target multiple cancer metabolic pathways [6]. Although immunotherapy shows promise, it encounters difficulties in improving response rates, particularly in breast cancer treatments [7]. Targeting reactive oxygen species (ROS) in tumor cells is a promising strategy which requires addressing issues related to ROS regulation for effective cancer therapy [8]. New technological developments such as deep learning-based systems offer potential solutions for enhanced early diagnosis and treatment planning [9]. While many challenges persist in the detection and treatment of cancer, a multidisciplinary approach, innovative solutions, and a focus on patient-centered care are necessary to overcome these challenges and improve cancer treatment outcomes.

Advances in cancer genomics have provided valuable insights into the genetic alterations that drive cancer development [10]. The classification of human cancers is based on factors such as the cell of origin and metabolic factors [11]. Large-scale genomic studies have revealed new features of cancer genomes, which can lead to the identification of potential biomarkers and therapeutic targets [12]. The importance of targeting specific mechanisms involved in cell division to impede tumor growth has been highlighted in recent studies [13]. Researchers have explored the metabolic reprogramming of cancer cells as a promising avenue for cancer therapy, including cell growth regulation through AMPK-PGC-1α-mediated metabolic switches [14]. The Yes-associated protein (YAP)-dependent Nupr1 pathway is a significant contributor to the development of tumor-repopulating cells with high tumorigenic potential [15]. YAP is a transcriptional co-activator oncoprotein that is associated with the Hippo pathway. YAP moves from the cytoplasm to the nucleus after gene transcription activation, and it plays a role in cell division and apoptosis and also influences aspects of cell proliferation, tumorigenesis, mechano-sensing, cell lineage, cell fate determination, and wound healing and regeneration [13]. Improved knowledge related to these, and associated, factors will have huge implications in understanding cancer progression and developing targeted interventions.

Biomimetic hydrogels are a type of smart material that imitate the natural extracellular matrix (ECM) found in tissues [16]. These hydrogels are made up of hydrophilic polymers that have the ability to absorb and retain large amounts of water, giving them similar softness and elasticity to biological tissues [16,17]. By engineering these hydrogels, they can possess properties that closely resemble those of the native ECM, such as mechanical strength, porosity, and bioactivity [16,17,18,19,20]. In the field of cancer therapy, biomimetic hydrogels have various applications, including drug delivery systems [21,22], tissue engineering scaffolds [23], and platforms for studying cancer cell behavior [23,24]. One of the mechanisms of action in cancer therapy is controlled drug delivery, where the hydrogels can encapsulate anticancer drugs and release them in a controlled manner in response to specific stimuli or over a prolonged period [25]. This controlled release helps in achieving localized drug concentrations, reducing systemic toxicity, and improving patient compliance [25,26]. Additionally, hydrogels can also be used in tissue engineering to serve as scaffolds for the growth of new tissue, which is particularly beneficial in reconstructive surgery after tumor removal [27]. They can also be utilized to create in vitro models that mimic the tumor microenvironment, aiding in the study of cancer biology and the development of new therapies [24,28]. Another application is immunotherapy, where certain hydrogels are designed to deliver immune-stimulating agents or present antigens to immune cells, activating the body’s own immune system to fight against cancer [29]. Lastly, specific hydrogels can be loaded with photosensitizers and used in photodynamic therapy (PDT), a treatment that utilizes light to activate a photosensitive drug, generating reactive oxygen species to kill cancer cells [30,31].

**Figure 1:**
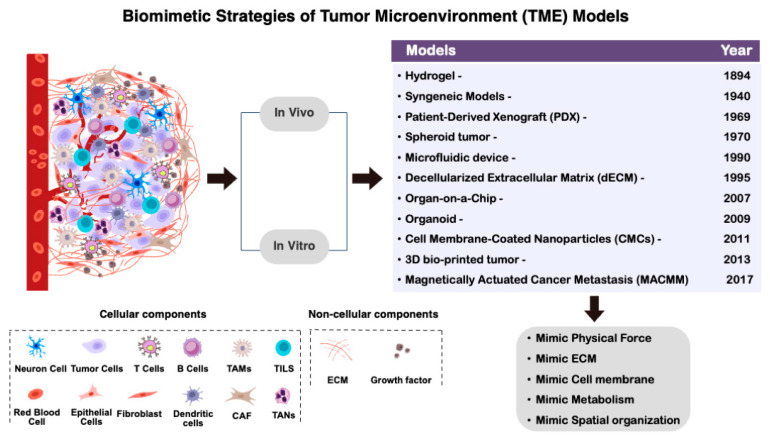
Schematic to represent the tumor microenvironment (TME) and biomimetic strategies of tumor microenvironment models: composition of the TME and cellular and non-cellular components (**right**); biomimetic strategies of tumor microenvironment model, in vivo and in vitro, associated with the biomimetic features in the TME (**left**).

It is crucial to have a deep understanding of cancer cell biology to develop effective treatment strategies that can adapt with cancer cells to remain effective. Efforts to understand fundamental cancer cell biology have been fruitful in enhancing our progress towards finding cancer cures. However, there is still much room for knowledge growth in these areas. One of the most promising avenues for studying cancer integrates key aspects of cell biology with inspired imitation in the area of adaptive biomimetic design strategies. Biomimetic strategies offer unique opportunities to design innovative therapeutic approaches that encompass various aspects of modeling cancer cell and tumor biology to discern the most effective diagnostic and treatment methodologies. When we couple these biomimetic ideas with hydrogel-based nanocomposites, great possibilities for synergistic interactions can arise that will provide groundbreaking insights for finding effective long-term cures (Figure 1). 

There is a broad scope of biomimetic approaches that can provide a rich space for exploration. Aspects of cellular organelle and molecular components, cellular processes such as division, metabolism, apoptosis, angiogenesis, and immune response are just a few of the many avenues that can provide fertile ground for discovery. In this review, we will connect biomimetic design strategies with unique bioinspired hydrogel systems to highlight strategic cancer therapies. A brief introduction to biomimetics through the lens of cell membranes, the extracellular matrix (ECM), and biological nanocomposite hydrogels will serve as a bridge to lead into a description of unique research ideas and trends that are unearthing some very innovative pathways to detect, model, and treat various forms of cancer. The next section will cover aspects of biomimetic design strategies followed by a focus on modeling cancer progression and etiology, cancer diagnostics, and cancer mechanobiology. The final section will highlight cutting-edge ideas related to Biomimetic Hallmarks of Cancer that are inspired by the classic Hallmarks of Cancer series [32,33,34].

## 2. Biomimetic Cancer Systems

### 2.1. Biomimetic Design for Cancer Therapy—Biomimetics and Biomimicry

Biomimetics comes from the Latin words bio (life) and mimere (to mimic) [35]. Biomimicry is an innovative design approach that takes inspiration from nature to address complex human challenges by observing and replicating phenomena exhibited by biological systems [36]. Imitation is indeed the highest form of flattery and nature provides a rich resource for exercising biomimicry. In healthcare, biomimicry contributes to developing advanced technologies and materials that imitate biological structures and processes for various medical purposes [37]. Researchers have successfully engineered nanomaterials for targeted drug delivery, cancer therapy, and tissue regeneration by mimicking natural structures and functions [38,39,40]. Biomimicry has been crucial in creating bio hybrids and artificial cells for cancer therapy [41]. Engineered bio hybrids have emerged as innovative platforms for delivering therapeutic agents for conducting synergistic cancer treatments [42,43]. Nanoparticles coated with cancer cell membranes have demonstrated the potential to enhance drug delivery efficiency and specificity to improve treatment outcomes [44]. The development of biomimetic carriers utilizing giant membrane vesicles has shown promise in targeted drug delivery and photodynamic/photo-thermal therapy for cancer treatment [45]. A novel method for creating magnetically propelled hydrogel particle motors uses ultrasound-assisted hydrodynamic electrospray ionization jetting, which allows for precise control over the size and shape of alginate-based particles loaded with magnetite nanoparticles, enabling targeted drug delivery with the potential to navigate through biological tissues under magnetic guidance [22]. The concept of biomimicry has found practical application in developing bio-functional biomaterials used in various biomedical fields [43,45,46]. These biomaterials draw inspiration from natural biological systems, and they are specifically engineered to cater to healthcare requirements in tissue engineering, drug delivery, and medical imaging [43]. Using biomimicry principles, scientists and researchers strive to create sophisticated materials that are biocompatible and possess specific functionalities tailored to medical needs. 

### 2.2. Biomimetic Drug Delivery Systems

Biomimetic applications in drug delivery systems have significantly advanced the field by utilizing nature-inspired designs to improve drug targeting, controlled release, and therapeutic efficacy by reducing side effects and advancing precision medicine [47]. These biomimetic carriers offer advantages in drug delivery by enhancing drug loading capacity, improving cellular uptake, and enabling site-specific drug release for effective treatment [39]. Biomimetic drug delivery systems are at the forefront of drug delivery innovation, providing a targeted and controlled release of therapeutic agents [26]. The emergence of biomimetic systems in cancer treatment represents a significant advancement in oncology [37]. The development of biomimetic nano-carriers for cancer-targeted therapy showcases the potential of nanotechnology in revolutionizing cancer treatment methods [39]. Biomimetic nanoparticles have emerged as a drug delivery platform for precise cancer therapy to enhance drug biocompatibility and specificity within the tumor microenvironment [48]. Additionally, the utilization of biomimetic delivery platforms for cancer vaccines has introduced a novel strategy for creating cell-derived delivery systems, thereby improving the effectiveness of cancer immunotherapy [49]. Biomimetic active materials have also shown potential in guiding immunogenic cell death (ICD) in cancer immunotherapy by boosting the immune response against cancer cells [29]. This can promote tumor cell death and enhance the overall efficacy of cancer immunotherapy [29,50]. These instances highlight the various uses of biomimetic systems in cancer therapy, ranging from investigating metastasis in organ-specific microenvironments to precise drug delivery for enhanced immune response. These systems can improve the pharmacokinetics and biodistribution of drugs, reduce side effects, and target drugs more effectively to diseased tissues. Here are some examples of biomimetic drug delivery systems and the drugs or types of drugs they can carry (Table 1).

### 2.3. Cell Membrane Mimetics

**Figure 2:**
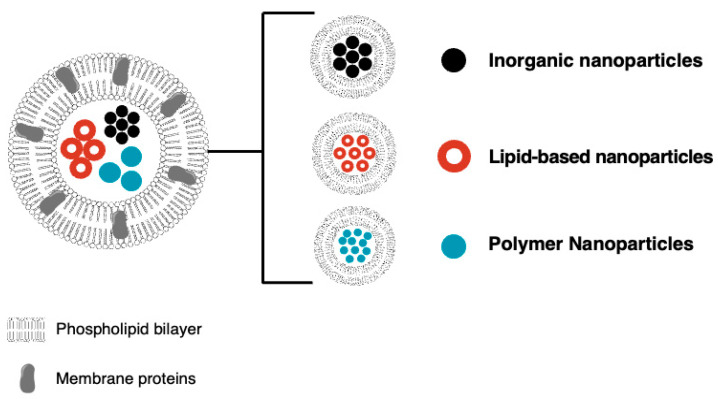
Cell membrane biomimetic nanoparticles consist of cell membrane coatings encapsulating different nanoparticles (NPs) for cancer therapy.

The cell membrane is a fascinating structure that is composed of a raft-like sheet of lipid macromolecules that are arranged in a double layer [44]. The characteristic appearance of the cell membrane is the result of hydrophilic polar head groups that are on either side of the membrane with the double phospholipid tails pointing inward to accentuate the hydrophobic character [70]. The cell membrane serves as a selectively permeable barrier that guards the cell by interrogating molecular entities that desire to pass into the cell [71]. If the molecules have the proper clearance as specified by selective factors, the sentry proteins that stand guard at the gates of the cell allow the molecules to pass through the cell membrane and gain entry into the cell [72]. Biomimetic approaches that utilize endogenous materials like cell membranes are designed to enhance drug delivery efficiency to improve targeting selectivity and reduce systemic toxicity by mimicking the surface properties of cells [73]. These systems replicate natural cell structures to enhance biocompatibility, increase drug delivery specificity, enhance circulation time and therapeutic efficacy, and minimize off-target effects [44,70]. This approach presents a tactic for improving the efficiency of inorganic nanoparticles to promote personalized treatments in cancer therapy (Figure 2) [31,72,74].

The scope of cell membrane-camouflaged nanoparticles encompasses normal and cancer cell membranes, blood–brain-barrier-penetrating albumin nanoparticles and bioengineered bacterial outer membrane vesicles for targeted drug delivery [26,75,76]. Biomimetic drug delivery systems based on red blood cells, platelet-derived extracellular vesicles, and cell membrane-coated nanoparticles have demonstrated potential in targeted drug delivery for cancer therapy and inflammatory diseases [77,78,79]. Nanoparticles coated with cancer cell membranes have demonstrated the potential to enhance drug delivery efficiency and specificity to improve treatment outcomes [44]. Erythrocyte membrane-cloaked nanoparticles specifically designed for starvation-activated colon cancer therapy can target hypoxic regions within tumors and effectively deliver therapeutic agents, such as glucose oxidase and antineoplastic agents [80]. Giant membrane vesicles and virosomes show promise for synergistic therapeutic applications and have been developed for targeted drug delivery and photodynamic/photo-thermal therapy [45,81]. Biomimetic nanoparticles functionalized with components from bacterial outer membranes have been created to facilitate drug delivery to the brain. These nanoparticles are designed to replicate how bacteria interact with endothelial cells to aid in the transportation of drugs across the blood–brain barrier for targeted therapy [82]. 

Cell membrane-camouflaged nanoparticles can also be utilized for cancer theragnostics by combining targeted therapy with integrated diagnostics [83]. The term theragnostics is a portmanteau that combines the Greek word gnosis (knowledge) with therapy and diagnosis. The term theranostics is more common. Theranostics specifically refers to radionuclide therapy that employs a pair of radiopharmaceutical agents containing radionuclides for diagnostic imaging and/or therapy [83,84]. Radionuclides can be attached to a vector, such as a small molecule, peptide, antibody, and/or nanoparticle, through a linker molecule [85]. Subsequently, the vector (ligand) can bind to cell surface receptors that are located within the cell membrane [84]. The cell membrane is leveraged to offer a versatile platform for targeting specificity and therapeutic efficacy in cancer treatment [83]. Biomimetic nanotechnology has been utilized to develop tumor-selective and tumor-specific near-infrared (NIR)-activated photo-nanomedicines for cancer therapy [86]. These nanomedicines leverage biomimetic approaches to enhance tumor-specific drug delivery and therapeutic efficacy, offering potential advancements in cancer treatment [86]. The biomimetic approach of cell membrane-coated semiconducting polymer nanoparticles has been explored for enhanced multimodal cancer photo-theranostics, highlighting the versatility and potential of these systems in targeting cancer cells [71]. A few specific examples include the use of biomimetic cell membrane-camouflaged nanoparticles in cancer phototherapy for improved drug delivery and treatment effectiveness [30], cell membrane-derived biomimetic nanotechnology for cancer immunotherapy and cutting-edge strategies for antitumor immune therapeutics [87], and studying the interaction between biomimetic nanoparticles and the immune system to fine tune the nano–bio interface in immunomodulation [88].

### 2.4. Biomimetic Hydrogel and Biomimetic Polymer Nanocomposites

Biological hydrogels are composed of a three-dimensional network of hydrophilic polymers that can absorb and retain large amounts of water [89]. This high water content allows them to closely mimic the natural environment of living tissues, making them biocompatible and suitable for various biomedical applications in targeted drug delivery, tissue engineering, and regenerative medicine, including cancer treatment, diabetes, and cardiovascular disease [90]. The unique network structure of biological hydrogels can facilitate the encapsulation and controlled release of drugs, growth factors, or other bioactive molecules to make biological hydrogels more valuable for drug delivery applications [91]. The customizable properties of hydrogels, including porosity, degradation rate, biocompatibility, high water content, unique three-dimensional network structures, self-healing properties, biodegradability, and swelling behavior, allow for the tailoring of drug release kinetics [16]. Researchers can achieve targeted and sustained drug delivery by integrating drugs into hydrogels to enhance therapeutic outcomes while minimizing side effects [92,93]. Some biological hydrogels exhibit self-healing properties, and this allows them to repair damage or undergo reversible changes in response to external stimuli [16]. This self-healing characteristic significantly enhances the durability and longevity of the hydrogels and makes them highly suitable for long-term applications in tissue engineering and drug delivery [94]. Many biological hydrogels are biodegradable and can be efficiently broken down and metabolized by the body over time. This property proves advantageous in applications where temporary support or controlled release is required [95].

There are currently no hydrogels specifically approved by the FDA for cancer therapy itself. However, hydrogels have been granted FDA approval as drug delivery devices and can be utilized to transport and release anti-cancer medications. For instance, Lupron Depot (PLGA copolymer) and Eligard (PLGA-based injectable implant) are both injectable hydrogels containing leuprolide acetate for treating prostate cancer [96]. These hydrogels work by suppressing testosterone production and slowing the growth of prostate cancer cells. Another FDA-approved hydrogel called SpaceOAR, which consists of hyaluronic acid, is used as a spacer during radiation therapy for prostate cancer [97]. It creates a temporary gap between the rectum and prostate gland, thereby reducing the risk of side effects [97]. Additionally, OncoGel (also known as ReGel/paclitaxel) is a thermosensitive hydrogel that is injected directly into tumors and contains the chemotherapeutic drug paclitaxel, which is slowly released over time [98]. This localized delivery method can help to reduce side effects associated with traditional chemotherapy [98]. Although these hydrogels have been approved by the FDA, they are not specifically intended for cancer treatment.

In the realm of cancer treatment, biomimetic hydrogels play a vital role in effectively delivering therapeutic agents to tumor sites [98,99]. pH- and enzyme-responsive hydrogels with self-healing properties have been developed to deliver anticancer drugs like gemcitabine for pancreatic cancer treatment [100,101]. These hydrogels efficiently guide the delivery of encapsulated drugs to tumor tissues to enhance treatment efficacy [100]. Injectable nanocomposite hydrogels have emerged as a promising avenue for cancer therapy [102]. These hydrogels consist of a polymer network with dispersed nanoparticles and possess excellent water-swelling capabilities and suitable mechanical properties [17]. Hybrid hydrogels that incorporate a decellularized skin extracellular matrix have been developed to enhance the physical and biological properties of fibrinogen hydrogels for skin bioprinting applications [103]. These advanced hydrogels improve cell viability and structural strength and open new possibilities for skin tissue engineering and cancer therapy [103].

pH-responsive and enzyme-responsive hydrogels can change their properties, such as swelling or stiffness, in response to changes in the surrounding acidity (pH) [104]. These materials, such as N-vinyl imidazole (NVIm) and 4-vinylpyridine (4-VP), exhibit responsive behavior to changes in pH [105]. NVIm and 4-VP possess charged groups that can be triggered by acidic and basic conditions, respectively [105]. This charge-induced swelling or shrinking of the hydrogel can be utilized to deliver therapeutic agents to cancerous tissues, change their stiffness, or even release encapsulated cargo depending on the specific pH [104,105]. Acrylic Acid (AAc) is another monomer with interesting properties. AAc has the ability to swell at higher pH levels, which makes it useful for the controlled release of encapsulated drugs. This swelling behavior is particularly advantageous in the slightly alkaline environment found in tumors [21]. On the other hand, Methacrylic Acid (MAAc), which is similar to AAc, also exhibits swelling and drug release at higher pH levels. However, MAAc-based hydrogels offer better stability compared to AAc-based hydrogels [21]. These unique characteristics of AAc and MAAc make them promising candidates for drug delivery systems in cancer therapy. The use of enzyme-responsive hydrogels can benefit cancer therapy by incorporating bonds that can be broken down by specific enzymes, such as hyaluronidase, an enzyme found in biological fluids [105,106]. These hydrogels allow for controlled drug release and can also change their own structure [101,107]. Additionally, enzyme-responsive hydrogels can be utilized in tissue engineering processes, where the scaffold’s degradation by cellular enzymes can promote cell growth and the deposition of matrix material [107].

The utilization of biomimetic polymer nanocomposites has expanded to include sensing applications [106]. Specifically, researchers have developed hemin/coordination polymer-based nanocomposites for biosensing purposes [106]. These nanocomposites aim to imitate the biological functions of heme within enzyme-like structures, highlighting the potential of biomimetic systems in the creation of functional materials for sensing and diagnostic applications [106]. Moreover, in the field of regenerative medicine, biomimetic polymeric nanocomposites have been investigated for their ability to control cell differentiation and facilitate tissue engineering [16,46]. Scientists have successfully enhanced the osteogenic differentiation of human mesenchymal stem cells by incorporating bioinspired elements to enhance tissue regeneration [108]. The biomimetic properties of these nanocomposites enable effective interaction with biological systems and offer customized solutions for tissue repair and regeneration. These examples highlight unique possibilities that may be designed and employed for treating tissues damaged by cancer based on these principles (Table 2).

### 2.5. ECM-Based Hydrogels

The dynamic and intricate microenvironment of the ECM is a sophisticated meshwork of proteins, glycoproteins, and proteoglycans that envelop and sustain cells within tissues [121,122]. The ECM acts as a reservoir for growth factors, cytokines, and other signaling molecules that profoundly impact and orchestrate cell behavior [123]. The sequestration and release of these bioactive molecules allows the ECM to play a critical role in regulating a myriad of cellular processes crucial for tissue development, maintenance of homeostasis, and progression of diseases including cell growth, differentiation, cell adhesion, migration, proliferation, and survival processes [124]. Beyond its structural function, the ECM functions as a dynamic microenvironment communicating with cells through diverse signaling pathways, thereby shaping their function and destiny [125]. A comprehensive understanding of the complex interactions between cells and the ECM is imperative for furthering our comprehension of cell biology and devising innovative therapeutic approaches for a wide array of health conditions [126]. Cell–ECM interactions are mediated by integrins [125,127]. Integrins are cell surface receptors that bind to ECM proteins and transmit signals that govern cellular behavior [127]. These interactions are indispensable for wound healing, tissue development, and the modulation of immune responses [128].

Alterations in the composition and arrangement of the ECM can promote tumor progression, invasion, and metastasis [129]. Dysregulation of matrix metalloproteinases (MMPs), enzymes responsible for degrading ECM components, is often observed in cancer and contributes to ECM remodeling, facilitating the migration and invasion of tumor cells [113,129]. The behavior and fate determination of stem cells are significantly impacted by the extracellular matrix (ECM) [130]. Stem cells engage with the ECM via integrin-mediated signaling pathways, which are responsible for governing their self-renewal, differentiation, and commitment to specific lineages. Both the biochemical and mechanical characteristics of the ECM are pivotal in steering stem cell behavior and facilitating tissue regeneration [131].

### 2.6. Biomimetic ECM Drug Delivery Systems

Mimicking the ECM in cancer treatment offers numerous advantages that can significantly impact the effectiveness of therapies [115]. Biomimetic strategies aim to imitate the structural and biochemical signals present in the natural ECM by recreating a microenvironment that closely resembles native tissues [132]. Scientists can provide a supportive environment for cancer cells to allow a more accurate simulation of tumor behavior and drug reactions [133]. Models based on a biomimetic ECM provide a platform for studying cancer progression and drug responses under conditions that closely resemble the body’s natural state [28]. Three-dimensional cancer models engineered with ECM components can replicate the complex structure of solid tumors, enabling the investigation of tumor development, invasion, and response to therapies [28,133,134]. These approaches can offer valuable insights into cancer biology and treatment approaches. Another advantage of mimicking the ECM in cancer therapy is the potential to develop targeted drug delivery systems. By incorporating ECM-mimetic elements into drug carriers, researchers can improve the precision and effectiveness of drug transport to tumor sites. Biomimetic delivery systems based on ECM components can enhance drug retention, release profiles, and targeting accuracy, ultimately improving therapeutic outcomes and reducing off-target effects. Additionally, the utilization of biomimetic ECM-based methods can significantly aid in the advancement of customized cancer treatments [28,92,135]. By customizing the composition and characteristics of ECM-mimetic frameworks to resemble the unique attributes of individual tumors closely, scientists can construct models that are specific to each patient, allowing for the testing of drug responses and the optimization of treatment strategies [92]. This personalized approach to cancer therapy holds immense potential in enhancing treatment outcomes and minimizing the occurrence of adverse effects [136].

The focus on mimicking key aspects of the ECM tumor microenvironment provides an exciting area of research in cancer therapy [115]. Colorectal cancer (CRC) is a prevalent form of cancer globally, with limited treatment options, especially for patients in advanced stages, due to the complex tumor microenvironment consisting of an ECM, cells, and interstitial fluids [137,138]. The tumor microenvironment undergoes matrix remodeling which involves changes in the composition of ECM components and biophysical properties such as stiffness and tension due to the actions of matrix metalloproteinases (MMPs) that crosslink or degrade the ECM [129]. Patient-derived grafts are valuable tools for predicting cancer treatment outcomes, as they maintain intact ECM architecture and stromal components [139]. Cell-generated contractile forces can induce rapid and irreversible changes in the density and structure of physiologically relevant extracellular matrices like collagen I and fibrin, within minutes. This observation showcases the dynamic nature of ECM architecture [140].

## 3. Biological Hydrogels in Cancer Biomimetic Systems

Biological hydrogels play a critical role in cancer biomimetic systems. They serve as a versatile platform for mimicking the extracellular matrix (ECM) and creating physiologically relevant environments for the study of cancer behavior and the development of innovative therapeutic strategies. Various types of biological hydrogels are utilized in cancer biomimetic systems. Natural collagen-based biomimetic hydrogels replicate the ECM of biological tissues and provide cues for cell attachment, proliferation, and differentiation [23,141,142,143]. These hydrogels offer a biomimetic microenvironment for cancer cells, enabling the investigation of cell behavior and response to therapeutic interventions. Another example is self-assembling peptide hydrogels [18], which serve as biomimetic ECMs for engineering 3D cell microenvironments in cancer research. These hydrogels replicate the biological and physicochemical properties of the ECM to facilitate the formation of cell constructs and precise oncology remodeling in cancers. Micro- and nano-fabricated hydrogels inspired by the ECM are crucial in regenerative medicine and tissue engineering [20]. They are vital in regenerating complex tissues and biological systems and provide a tailorable biomimetic platform for cancer therapy. Matrigel is a naturally derived biomimetic hydrogel matrix that exhibits ECM-like biological properties. Matrigel has been used to investigate cell–ECM interactions and drug resistance in epithelial ovarian cancer cells [135]. Biomimetic hydrogels with self-protective functions can be utilized as artificial ECMs to encapsulate cells by forming a dynamic, flexible hydrogel network [110].

### 3.1. Hydrogel Tissue Mimetics

Biological hydrogels are crucial in imitating the ECM and facilitating cell behavior in various biomedical applications. These hydrogels create a biomimetic setting that closely resembles the microenvironment of natural tissues, providing a platform for cell growth, division, and differentiation. The exceptional properties of hydrogels, including biocompatibility, adjustable stiffness, and responsiveness to stimuli, make them excellent candidates for developing ECM-mimicking environments for cell culture and tissue engineering purposes [89,144]. The adjustable stiffness of hydrogels allows researchers to modify the mechanical properties of the matrix to match those of specific tissues or organs. By manipulating the crosslinking density or composition of the hydrogel, it becomes possible to create environments that closely imitate the stiffness of native tissues, providing cells with a supportive substrate for attachment, division, and differentiation [145]. Hydrogels can be designed with stimuli-responsive characteristics, enabling them to alter their mechanical attributes in response to external cues like pH, temperature, or light. This responsiveness grants dynamic control over the stiffness of the hydrogel, which can influence cell behavior and function [17,19,146,147]. Their capacity to mimic the structural and mechanical characteristics of natural tissues, along with their compatibility with living organisms and responsiveness to stimuli, renders them invaluable in constructing biomimetic settings that enhance cell proliferation, tissue regeneration, and therapeutic results.

### 3.2. Hydrogels and 3D Tumor Models

Hydrogels are used to construct 3D tumor models replicating the tumor microenvironment, including cell–cell and cell–matrix interactions. These models offer a platform for studying tumor biology, drug responses, and disease progression in a physiologically relevant context. Cells housed within hydrogels can interact with their surroundings, proliferate, and differentiate, closely resembling in vivo conditions. Adjustable stiffness and bioactive components of hydrogels can influence cell behavior, making them valuable tools for tissue engineering and regenerative medicine applications [18]. By introducing tumor cells into hydrogels, researchers can assess drug efficacy, tumor invasion, and metastatic potential in a controlled in vitro environment [22,148]. Overall, hydrogels serve as versatile platforms for drug delivery, cell encapsulation, and the development of 3D tumor models, making them invaluable in biomedical research for studying disease mechanisms, evaluating drug candidates, and advancing personalized medicine.

### 3.3. Clay-Based Hydrogels

Clays provide another great platform for designing biomimetic-based treatment platforms. Clays consist of a family of layered aluminosilicate minerals that display various morphologies ranging from the hexagonal plate-like physical form of China clay kaolin to halloysite rolled tubes [149]. The rolled-tube morphology of halloysite is reminiscent of microtubules, a very important cell cytoskeletal component that plays a key role in cell movement, cell division, and protein transport. The unique physicochemical properties of clays hold great promise in cancer treatments. They can induce apoptosis, adsorb and adhere to tumors, limit metastatic potential, and serve as delivery vehicles for cancer drugs. Simple adsorptions can be used as the primary delivery mechanism. Additional types of clays that show promise for contribution to cancer therapies include novel clay-based hydrogels [99,100], the class of synthetic clays called layered double hydroxides (LDHs) [150], and unique clay systems that exhibit self-gelling behaviors due to surface charge interactions [151]. LDHs possess the fascinating ability to intercalate various molecules within their interlayer space, such as cancer drugs, for a controlled delivery and release system [152,153].

## 4. Engineering Biomimetic Models for Mechanobiology of Tumor Progression

### 4.1. Modeling Cancer Metastasis

Metastasis is one of the “Hallmarks of Cancer” [154] and is responsible for most cancer-related deaths [155]. Metastasis occurs when cancer cells have the ability to invade the tissue surrounding the tumor stroma and move to other organs [140]. Cancer metastasis can be modeled as a multistep process that involves five main steps: (i) tumor cell invasion and local infiltration into the adjacent tissue, (ii) transendothelial migration of cancer cells into vessels (intravasation), (iii) survival in the circulatory system, (iv) extravasation (exit from blood vessels), and (v) colonization (subsequent proliferation in competent tissue and organs). The “seed and soil” hypothesis for cancer spread was proposed by Stephen Paget in 1889 after studying autopsy reports of women who died from breast cancer. Cancer cells are correlated with plant seeds, as they are widely disseminated via the blood stream for metastatic cancer cells (wind for plant seeds), but only grow when the cancer cells land in the tissues and organs with the right organ microenvironment (fertile soil for plant seeds). Cancer therapeutics may target the cancer cells or modulate the tumor microenvironment in ways that hinder key aspects of the metastasis [154,156].

Cancer cell mechanobiology studies focus on how the mechanical properties and chemical composition of the extracellular matrix (ECM) influences cancer cell metastasis. Cancer migration is crucial for several physiological processes including tissue morphogenesis, immune cell trafficking, wound repair, and metastasis. Cancer mechanobiology studies provide evidence for the design of targeted strategies to help impair the dissemination of cancer cells from the initial tumor to form metastases in other organs or tissues of the human body. Data from these studies can be used to build models to help explain how forces related to pressure, tension, and fluid flow and mechanical properties (stiffness and elasticity) affect cellular function and tumor progression. In addition, integrating nanocomposite biomaterials to investigate their interaction with cancer cells and the forces that are exerted both inside and outside of the biological system of cancer metastasis would equip researchers with additional data to develop more targeted therapeutic and diagnostic tools and techniques. In this section, we discuss cancer metastasis models that use biopolymer nanocomposites to administer controlled mechanical stress fields to cancer cells and their surrounding tumor stroma microenvironment.

### 4.2. Mechanobiology in Cancer Metastasis

Recent advances in engineering and the physical sciences have uncovered critical roles of the mechanical and structural properties of cells and tissues in guiding malignancy and metastasis. Tissue architecture and the mechanical properties of tissues and cells contribute to cancer progression. Dynamic remodeling of the stromal collagen network is one of the hallmarks of tumor progression. Cancer cells exert multiple forces against their surrounding environment, and this presents a complex system for analysis [157].

Biomimetic design principles of nanocomposite hydrogels focus on mechanical properties to replicate extracellular matrix (ECM) stiffness. A recent study demonstrated that extracellular matrix (ECM) stiffness influences DNA methylation levels by facilitating the PKCα-dependent nuclear translocation of DNMT3L. This sheds light on how cells sense mechanical cues from the ECM to regulate gene expression during cell–ECM interactions [158]. Another study investigated the vinculin proline-rich linker region’s interaction with vitexin α in detecting changes in ECM stiffness to understand molecular responses to ECM stiffness [159]. This underscores the significance of comprehending the molecular mechanisms responsible for sensing ECM stiffness. The ability to quantify alterations in the local apparent stiffness of the ECM induced by magnetic forces underscores the essential role of ECM stiffness in modulating cell–matrix adhesion, focal adhesion dimensions, and cellular tension [24,160]. Stiffness-dependent regulation of Vinculin behavior has been investigated to explore how vinculin’s association with the actin cytoskeleton regulates its behavior in response to ECM stiffness variations. Their findings highlight the crucial role of actomyosin-generated forces in ECM stiffness sensing [161]. This research underscores the potential of nanocomposites in enhancing mechanical properties, offering insights into designing biomimetic materials custom-made to mimic ECM stiffness. These studies can provide valuable insights regarding the design principles of polymer nanocomposites used in biomimetic applications. The main focus is on customizing the mechanical properties to imitate the stiffness of the extracellular matrix (ECM). By comprehending the molecular mechanisms and interactions that are responsible for sensing ECM stiffness, scientists and researchers can create nanocomposites that possess improved mechanical properties. These nanocomposites can then be utilized in various applications such as tissue engineering, regenerative medicine, and mechanobiology.

MDA-MB-231 is an invasive human breast cancer cell line that is widely used for in vitro studies of breast cancer metastasis. This cell line typically has poor metastatic tumorigenic potential [162]. However, this cell line becomes the basis for developing metastatic models for in vitro studies after the induction of genetic changes [163]. Breast cancer cells are influenced by collagen matrix alignment and redirect their migration via contact guidance [130,164,165]. Breast and prostate cancers and many other cancer types are mechanically sensitive [166,167]. Enhanced stiffness of fibroblast-seeded collagen gels stretched between PDMS micro-posts was demonstrated following tissue actuation at 2 Hz using a Nickel sphere and magnetic tweezers. The independent contributions of cells and matrix to the micro-tissue stiffness were decoupled by disrupting cell–matrix adhesions, disrupting the actin cytoskeleton, and killing the cells [168]. Alshehri et al. [24] designed and developed a unique model system to study the mechanobiology of tumors using breast cancer cells. The magnetically actuated cancer metastasis model (MACMM) used magnetic nanoparticles encased within mm sized chitosan alginate gel beads to deliver a tailored stress/strain profile to breast cancer cells embedded in a collagen matrix. Key results from the study suggested that a magnetic approach to actuate collagen hydrogel constructs with in situ frequency and magnitude-controlled mechanical stimulation delivery to breast cancer cells offers a unique method to address questions related to cancer cell and tumor mechanobiology and metastasis. The link between cancer response to mechanical loading in the stroma and disease outcome is not entirely clear. In vitro cancer cell tissue constructs with in situ force loading can help to clarify the role of mechanical forces in altering functional cancer cell behavior.

### 4.3. Cancer Cell–ECM Interaction

One of the known hallmarks of cancer is the alteration in cell–ECM interactions, which leads to induced cell migration and invasion [169]. The directional motion of cancer cells is controlled by several factors including Haptotaxis (the adhesive characteristics of the ECM substrate) [165], Chemotaxis (chemical concentration gradients) [166], Durotaxis (mechanical stimuli transmitted via ECM rigidity) [170,171], and Topotaxis (gradients of the nanoscale topographic features of ECM) [172]. Adherent cells can sense the mechanical properties of their surroundings by exerting a contractile force that can influence cell–matrix and/or cell–cell adhesions. ECM rigidity regulates cancer cell growth and cellular phenotype [173,174]. For example, breast cancer cells alone can increase the local fiber density of reconstituted collagen matrices by more than 150% [175]. Malignant tumors cause strain on the surrounding tissue, which changes the ECM stiffness gradient and can cause cancer cells to migrate [176]. The stiffness gradient of the ECM is controllable, yet not in the same platform to introduce the cells to more complex tissue constructs [177]. Thus, the interaction between the cells and the extracellular matrix remains unclear. Several transmembrane and scaffold microenvironment assays can be designed to study cell migration and mimic tumor niche characteristics in response to a chemical stimulus and matrix rigidity [178]. This is because the ECM plays an essential role in the regulation of many biological pathways that cause diseases [179].

ECM topographies influence cell adhesion, morphology, and migration, and have been used as a tool to study cell mechanobiology effects [180,181,182]. Several techniques such as 3D photolithographic patterning, collagen self-assembly, and tissue-mimetic platform aligned under an external magnetic field in the presence of MNPs have been reported to recreate 3D topography [109,182,183]. Many reports have focused on the role of microscale ECM topographies in modulating cellular adhesion [180], morphology [183], and orientation of cell migration through contact guidance [184]. Substrate topography provided by a fabricated ECM can impact the organization, arrangement, and distribution of integrin, dependent on cell type [172,180,185]. For example, focal adhesion kinase (FAK) and Ras-related C3 botulinum toxin substrate (Rac1) signaling provide directional guidance to MDA-MB-231 cell migration in 3D matrix alignment by reducing cell distribution speed and the anisotropy [165]. Matrix microstructure, composition, and contact guidance are the most influential external physical cues that transmit to intracellular cues. These signals can lead to a more fundamental understanding of physiological and pathological disease changes driven by ECM variation.

Malignant tumors produce compressive pressure on the surrounding tissue, leading the cells to migrate along stress-induced contour lines along the ECM boundaries [176,186]. Intra-tissue compression drives cells to migrate individually or as collective mass of cells whose movement depends on the force direction [186]. However, cell membrane protrusion formation is the initial stage that drives the directional movement of cancer cells [165]. Gu et al. [169] described this cell membrane protrusion as invadosome-like protrusions (ILPs), where directional mesenchymal cell invasion in vivo is a stimulated event regulated by cytokines, chemokines, and types of extracellular matrices (ECMs). Determination of the cell membrane mechanisms that drive protrusion formation is essential in furthering our understanding of cancer cell migration and metastasis [187]. A study by Boggs et al. [188] provided additional insight into the subcellular mechanisms at the microtubule level that contribute to cell membrane protrusions. The acetylation of lysine 40 in Alpha-tubulin promoted the generation of micro-tentacle-like cell protrusions, tumor cell reattachment, and chemotaxis. These characteristic features provide selective advantages for metastatic potential, which was particularly enriched in basal-like breast cancers. This provides an interesting opportunity for α-tubulin acetylation to serve as both a diagnostic and therapeutic target for metastatic breast cancer.

Speed and directional persistence are the main physical parameters to identify cancer cell migration [130,165,166,172,184,189]. Therefore, studying these two parameters (speed and directional cell migration) is a key to obtaining a more thorough understanding of cell–matrix interactions and the contact guidance of cells in various ECM microstructure alignments induced by magnetic field and beads [24,114]. As the extracellular matrix develops more alignment, persistence, and cell elongation along the alignment axis, it increases to display a higher cell projected area and dynamic changes in cellular morphology, which depend on the influence of focal adhesion kinase (FAK) and the Rac1 signaling [165].

**Figure 3:**
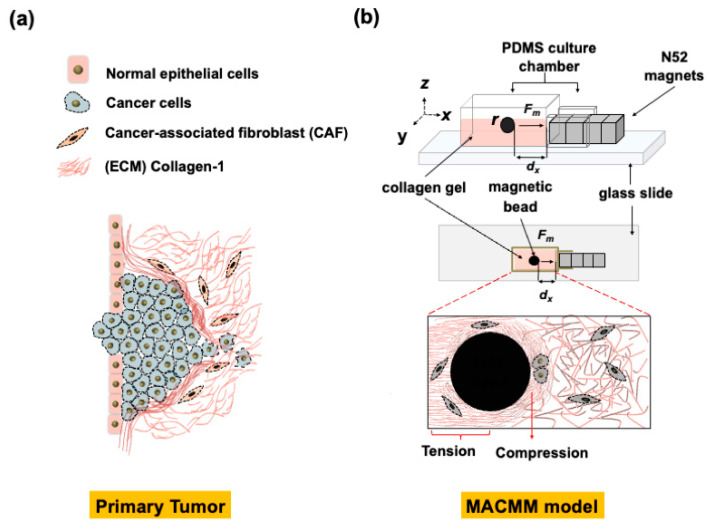
Comparison between primary tumor and MACMM model: (**a**) schematic representation of a primary tumor microenvironment structure, (**b**) the magnetically actuated cancer metastasis model (MACMM) provides the ability for microstructural alterations involving compression, tension, and shear on the collagen network to mimic the in vivo micromechanical environment surrounding a tumor.

Engineering approaches to study cancer cell mechanobiology are represented in controllable input parameters (i.e., ECM stiffness, composition, intra-tissue strain, and ECM network alignment) introduced to the cells. Quantitatively, the outcome response of cell measures (i.e., migration speed, migration phenotype, proliferation, and cell–ECM signaling) help determine the cell behavior in response to these controllable inputs. Engineering approaches can be used to manipulate and modulate cancer cells’ responses to multiple factors based on cause-and-effect interactions. As we gain more understanding of tumor biology following this approach, the role of the microenvironment is expected to take center stage in strategies to control tumor initiation, progression, and metastasis, in addition to the integral role of biomaterials, as we aim to alter the stroma to understand key aspects of cancer progression. Biomimetic models such as the MACMM model provides the ability for controlled microstructural alteration involving compression, tension, and shear on the collagen network, mimicking the in vivo micromechanical environment surrounding a tumor (Figure 3) [24]. There are tumor-associated collagen signatures, such as increased density, circumferential alignment, and perpendicular alignment to the cancer tumor, that may arise from specific internal force loading on the tumor stroma. The tissue culture of the model in vitro may help to explain how tumor-associated collagen signatures form.

## 5. Future Directions and Trends

The Hallmarks of Cancer provided a strong foundation for understanding cancer progression by addressing key questions related to the fundamental drivers for cancer [32]. One of the most important impacts of this groundbreaking work is that it provided a framework that clearly and concisely defined the motivating factors for cancer. The Hallmarks of Cancer provided a primer for understanding cancer fundamentals. Many of the answers to the questions that have been asked throughout history regarding the etiology of cancer cell genotypes were summed up in six (6) parameters that identified the areas where cancer cells alter normal cell physiology and collectively dictate malignant growth by evading the intrinsic safety features of normal cells. These parameters involve (1) self-sufficiency in growth signals (sustaining proliferative signaling), (2) evading growth suppressors (insensitivity to growth-inhibitory (antigrowth) signals), (3) evasion of programmed cell death (apoptosis—resisting cell death), (4) limitless replicative potential (enabling replicative immortality), (5) sustained angiogenesis (inducing angiogenesis), and (6) tissue invasion and metastasis (activating invasion and metastasis). Hanahan and Weinburg [33] expanded the Hallmarks to include two new factors: reprogramming of energy metabolism and immune system evasion to avoid destruction. The most recent addition to the Hallmarks [34] focuses on four (4) additional factors that are being elucidated. These factors are (1) unlocking phenotypic plasticity, (2) non-mutational epigenetic reprogramming, (3) polymorphic microbiomes, and (4) senescent cells.

Biomimetic systems have shown great promise in cancer therapy. Some systems have been developed to replicate the tumor microenvironment and recapitulate the interactions between cancer cells and surrounding tissue. This provides valuable insights for cancer studies [37]. Techniques such as biomaterial-based co-culturing, microfluidics, and organ-mimetic chips have been utilized to gain a better understanding of the interactions between metastatic breast cancer cells and the stroma at metastatic sites to gain insights about cancer progression and treatment [74]. Biomimetic strategies have taken on an enhanced level of sophistication by employing patient-derived organoids to recreate organ- and patient-specific microenvironments to study breast cancer metastasis [99].

There are a host of biomimetic-based strategies to fight cancer that are in various stages of discovery and development. The future looks bright for finding broad-based treatments that can lead to a once-and-for-all cure to this perplexing malady. The final section of this paper will focus on a few particularly intriguing approaches for treating cancer including the Hallmarks of Cancer and unique derivations that can be classified as Biomimetic Hallmarks of Cancer. Biomimetic Hallmarks of Cancer provides new revelations regarding the Hallmarks of cancer in terms of biomimetic lessons based on wound healing, metabolic factors, and the inverse acid profile of the tumor microenvironment and implications for cancer therapies. A final view of crab-based cancer therapies provides a uniquely promising platform for treating cancer and brings us full circle to close the loop from the initial comparisons between cancer and crabs to the use of crab components to fight cancer.

### 5.1. Biomimetic Hallmarks of Cancer

The Hallmarks of Cancer provided a cogent distillation of key factors which made it possible to envision a systematic approach to combating cancer through focused interventions. In essence, the Hallmarks paved the way for the development of biomimetic design strategies to combat cancer because it brought the key physiological factors that differentiate cancer cells into clear focus. This enabled further development of models, diagnostic tests, and therapies to harness biomimetic concepts and apply them towards understanding and treating cancer from the key vantage point of biomimetics. This approach facilitates a new framework for understanding cancer that can be described under the umbrella of Biomimetic Hallmarks of Cancer. The biomimetic approach extends the reach, effectiveness, and scope of the Hallmarks of Cancer. The following topics are a few that highlight a comparative pairing of cancer with other biological phenomena to form powerful analogies: Hallmarks of Cancer and Metabolism [190], Hallmarks of Cancer and Metastasis (revisited) [154], Hallmarks of Cancer and Wound Healing [191,192], and Hallmarks of Cancer and Acidosis of the Tumor Microenvironment [193,194].

#### 5.1.1. Hallmarks of Cancer and Metabolism

Pavlova and Thompson [190] proposed a new framework for the Hallmarks of Cancer based on key aspects of cancer cell metabolism. Their Six Hallmarks of proposed cancer-associated metabolic changes are (1) deregulated uptake of glucose and amino acids, (2) use of opportunistic modes of nutrient acquisition, (3) use of glycolysis/TCA cycle intermediates for biosynthesis and NADPH production, (4) increased demand for nitrogen, (5) alterations in metabolite-driven gene regulation, and (6) metabolic interactions with the microenvironment. Metabolic effects associated with cancer involve a paradoxical shift from glycolysis to fermentation for energy production which is known as the Warburg Effect reported that tumors consume extreme amounts of glucose relative to normal tissues [195]. Surprisingly, he found that the majority of the glucose was fermented to lactate rather than oxidized via normal respiration. The Nobel Prize was awarded to Otto Warburg in 1931 for his discovery and subsequent research related to the metabolic characteristics of cells. This propensity of cancer cells to shift from cellular respiration to fermentation to supply energy needs is a characteristic feature that can be utilized for therapy development [193,194].

#### 5.1.2. Hallmarks of Cancer and Wound Healing

There are unique similarities between physiological and cellular processes involved in cancer and wound healing. This connection provided additional grounds for exploring specific strategies to “heal” cancer akin to how wounds heal. The authors of [192] highlighted the keen observation that tissue repair and cancer share cellular and molecular processes that are regulated in a wound but misregulated in cancer. They proposed eight prospective hallmarks that might apply to both cancer and wound healing: (1) avoiding immune destruction, (2) wound-promoting inflammation, (3) activating invasion and migration, (4) inducing angiogenesis, (5) resisting cell death, (6) sustaining proliferative signaling, (7) evading growth suppressors, and (8) deregulating cellular energetics.

#### 5.1.3. Hallmark of Cancer and Acidosis of the Tumor Microenvironment

One of the fascinating repercussions related to metabolic studies on cancer tumors is that, in contrast to normal cells, cancer cells prefer an acidic environment. pH values near 6.7 promote cancer cell growth, whereas pH 6 and below and moderate to extreme basic pH in the range 8.4–9.2 retard cancer cell growth and favor a cell-growth-model shift away from G0/G1 [193]. This observation holds promise for developing additional targeted therapies using the biomimetic model for understanding how pH influences the tumor microenvironment.

### 5.2. The Gut Microbiome and Halalmarks of Cancer

We can even express additional creativity in coining a new category of Cancer Hallmarks. The “Halalmarks” of Cancer are based on food-, diet-, and microbiome-based interventions for the treatment and rehabilitative prevention of cancer [196]. The concept of prehabilitation involves proactively preventing problems from happening [196,197]. Halal is a term that originates from the Arabic language, is deeply rooted in Islamic law, and embodies ancient health practices that are highlighted in the writings and practices of many major religions (Kosher in other religions) and is still timely for today [198]. There is a host of food-based interventions in terms of foods to avoid (such as excess sugar and some red meats) and foods to eat in abundance (fresh fruits and vegetables, especially green vegetables such as broccoli and kale). The gut microbiome holds a treasure trove of therapies based on its impact on health and its keen relationship with what we eat [199]. Prebiotic components of our diet, such as fiber, provide the raw materials that are used to produce short-chain fatty acids, which play key roles in health. One unique role that short-chain fatty acids can play in health promotion includes the prevention of metastasis of colorectal cancer (CRC) cells and inducing apoptosis in many types of cancer cells while not affecting normal healthy cells [200].

The gut microbiome plays a role in antitumor immune response, but the specific mechanisms are still under investigation. A recent study has identified a Rhamnose-rich polysaccharide (RHP) produced by a food commensal microbiome species strain of Lactiplantibacillus plantarum (LP) that limits tumor growth and promotes antitumor immunity by inducing tumor-associated macrophages to sequester excess iron [201]. LP exhibits promise for the development of a new multi-faceted line of ‘oncobiotics’ based on cancer therapies [202]. The mechanism is further elucidated to involve a two-pronged approach. The first interaction involves skewing the tumor-associated macrophage population to a classically active phenotype along with the generation of a sustained CD8^+^ T cell response. The second interaction triggers ‘nutritional immunity’ in macrophages to deploy the high-affinity iron transporter lipocalin-2 to capture and sequester iron in the tumor microenvironment. This process induces a cycle of tumor cell death, epitope expansion, and subsequent tumor clearance.

### 5.3. What Is in a Name? The Etymological Origin of Cancer

Hippocrates, known as the father of modern medicine, used the Greek terms carcinos (non-ulcer forming) and carcinoma (ulcer forming) to describe tumors. These words reference crabs and are reminiscent of crabs due to the spider-like projections that cancers often display. Celsius is credited with translating the terms to Latin, from which we have the word cancer. The creative connection between cancer and crabs has been crafted into a series of promising potential biomimetic-based treatment protocols for cancer based on crab biochemistry and crab shell components. Horseshoe crabs have a long history of providing natural resources to benefit people. Horseshoe crabs were used as a rich source of fertilizer to support agriculture. In recent times, the blue blood from horseshoe crabs has been harvested on an annual basis and used to make the limulus test, which is a very important assay for bacterial infections [203]. One particular species of horseshoe crab, the Mangrove Horseshoe Crab from South Asia, produces a powerful neurological toxin (tetrodotoxin) that has been used clinically to treat cancer pain and heroin withdrawal pain. A new class of host defense peptides from the horseshoe crab Tachypleus tridentatus have received attention for developing anticancer therapy. The host peptide Tachyplesin-1 (T1) exhibits unique anticancer behaviors based on methods to defeat invading microbes by piercing cell membranes in a manner analogous to how a sharp needle pierces a water balloon. Scientists, in their search for a comparable biomimetic technique to target and pierce cancer cell membranes selectively, have discovered that T1 can be made into a circular peptide by binding the ends together. This structurally modified cT1 displays similar anticancer properties but has lower hemolytic properties and higher stability. The hydrophobicity and charge of cT1 analogues influence membrane binding affinity and cytotoxicity. Two specifics analogues exhibited increased selectivity for melanoma cells and one analogue could enter the cancer cell with lower toxicity and higher efficacy, without damaging the membrane structural integrity [204]. This shows that we have come full circle in progressing from early attempts to classify cancer based on similarities with crabs to the development of promising treatments based on crab biology.

## 6. Conclusions

Biomimetic design principles can be used to enhance the effectiveness of hydrogels for the modeling and treatment of various aspects of cancer. This review provides a broad survey of a few areas that hold promise for advancing our understanding of cancer by viewing biomimetic hydrogels to study cancer therapies and mechanisms related to mechanobiology effects. Biomimetic systems have shown great promise in cancer therapy. Some systems have been developed to replicate the tumor microenvironment and recapitulate the interactions between cancer cells and surrounding tissue. This provides valuable insights for cancer studies. Techniques such as biomaterial-based co-culturing, microfluidics, and organ-mimetic chips have been utilized to gain a better understanding of the interactions between metastatic breast cancer cells and the stroma at metastatic sites to gain insights about cancer progression and unique treatments employing patient-derived organoids to recreate organ- and patient-specific microenvironments. There are a host of biomimetic-based strategies to fight cancer that are in various stages of discovery and development. The future looks bright for finding broad-based treatments that can lead to a once-and-for-all cure for this perplexing malady.

## Figures and Tables

**Table 1:** Biomimetic drug delivery system for cancer therapy.

Biomimetic Drug Delivery System	Drug Name	Ref.
Liposomes	Doxorubicin (Doxil)	[51,52]
	Daunorubicin (DaunoXome)	[53]
Polymeric Micelles	Paclitaxel (Genexol-PM)	[54,55]
Dendrimers	Cisplatin, Methotrexate	[56]
Viral-like Particles (VLPs)	Nucleic acids for gene therapy	[57]
Exosomes	Chemotherapeutics, Proteins	[58,59,60]
Cell-Penetrating Peptides (CPPs)	Various drugs and biomolecules	[61]
Biomimetic Nanoparticles	Imaging agents, Chemotherapeutics	[62]
Biomimetic Membranes	Antibiotics, Bioactive molecules	[63,64]
Biomimetic Enzyme Mimetics	Prodrug activators	[65]
Biomimetic Scaffolds	Growth factors, Antibiotics	[66,67]
Biomimetic Prodrugs	Capecitabine	[68]
Biomimetic Surface Coatings	Drug-eluting stents (e.g., sirolimus, paclitaxel)	[25,69]

**Table 2:** Biomimetic applications of polymer nanocomposites for cancer therapy.

Biomimetic Application	Key Functionality	Inspiration from Nature	Key Properties of Polymer Nanocomposites	Example	Ref.
Drug Delivery	Controlled Release, Targeting	Drug-carrying viruses, Release based on specific stimuli	Biocompatibility, Degradation, Targeting ligands	Stimuli-responsive polymer nanocomposites for targeted drug delivery to cancer cells (e.g., pH-sensitive polymers releasing drugs in the acidic tumor microenvironment)	[76]
Tissue Engineering	Cell Adhesion, Proliferation, Differentiation	Extracellular matrix, Scaffolding for tissue growth	Biocompatibility, Mechanical properties (Mimicking target tissue), Controlled porosity	Biomimetic scaffolds mimicking natural bone structure for bone regeneration (e.g., scaffolds with interconnected pores mimicking bone)	[109]
Biosensors	Biorecognition, Signal Transduction	Biological receptors, Enzyme–substrate interactions	Biocompatibility, Selectivity, Sensitivity	Nanocomposite-based sensors for glucose detection mimicking taste receptors (e.g., incorporating enzymes that react with glucose)	[40]
Gene Delivery	Efficient Gene Delivery, Controlled Release	Viral vectors for gene transfer	Biocompatibility, Low immunogenicity, Controlled release of genetic material	Polymer nanocomposites for siRNA delivery to silence genes involved in diseases (e.g., using cationic polymers to complex with negatively charged siRNA)	[110]
Implants	Improved Biocompatibility, Enhanced Osseointegration	Natural bone structure, Load-bearing capacity	Biocompatibility, Mechanical strength (Mimicking bone), Osseoconductive properties	Biomimetic hydroxyapatite-based implants for improved bone bonding (e.g., mimicking the mineral component of bone)	[111,112,113,114]
Antibacterial Coatings	Bacterial Adhesion Inhibition, Biofilm Prevention	Antimicrobial peptides on insect wings	Biocompatibility, Antimicrobial activity, Surface modification for long-lasting effect	Polymer nanocomposite coatings incorporating natural antimicrobial peptides to prevent bacterial infections on medical devices	[115]
Wound Healing Dressings	Enhanced Healing, Reduced Inflammation	Skin barrier function, Moist wound environment	Biocompatibility, Controlled drug release, Biodegradability	Biomimetic wound dressings mimicking the skin’s barrier function while promoting healing and reducing inflammation	[116]
Hemostatic Materials	Blood Clotting Acceleration, Reduced Bleeding	Platelet aggregation, Blood clotting cascade	Biocompatibility, Hemostatic activity, Shape conformity	Polymer nanocomposites mimicking the structure and function of platelets to accelerate blood clotting at wound sites	[117,118,119]
Biomimetic Enzymes	Targeted Enzyme Therapy, Enhanced Biocatalysis	Natural enzymes with high efficiency and specificity	Biocompatibility, Enzyme immobilization, Controlled activity	Polymer nanocomposite-based artificial enzymes mimicking natural enzymes for targeted treatment of diseases	[38,120]
Biomimetic Hydrogels	Tissue Regeneration, Drug Delivery	Extracellular matrix, Natural hydrogels	Biocompatibility, Biodegradability, Tunable mechanical properties	Biomimetic hydrogels mimicking the properties of the extracellular matrix for tissue engineering and controlled drug delivery	[16,27,46]

## Data Availability

Not applicable.
